# Magnetoencephalography Study of Right Parietal Lobe Dysfunction of the Evoked Mirror Neuron System in Antipsychotic-Free Schizophrenia

**DOI:** 10.1371/journal.pone.0028087

**Published:** 2011-11-22

**Authors:** Yutaka Kato, Taro Muramatsu, Motoichiro Kato, Yoshiyuki Shibukawa, Masuro Shintani, Masaru Mimura

**Affiliations:** 1 Department of Neuropsychiatry, Keio University School of Medicine, Tokyo, Japan; 2 Laboratory of Brain Research, Oral Health Science Center, Tokyo Dental College, Chiba, Japan; 3 Department of Physiology, Tokyo Dental College, Chiba, Japan; Chiba University Center for Forensic Mental Health, Japan

## Abstract

**Introduction:**

Patients with schizophrenia commonly exhibit deficits of non-verbal communication in social contexts, which may be related to cognitive dysfunction that impairs recognition of biological motion. Although perception of biological motion is known to be mediated by the mirror neuron system, there have been few empirical studies of this system in patients with schizophrenia.

**Methods:**

Using magnetoencephalography, we examined whether antipsychotic-free schizophrenia patients displayed mirror neuron system dysfunction during observation of biological motion (jaw movement of another individual).

**Results:**

Compared with normal controls, the patients with schizophrenia had fewer components of both the waveform and equivalent current dipole, suggesting aberrant brain activity resulting from dysfunction of the right inferior parietal cortex. They also lacked the changes of alpha band and gamma band oscillation seen in normal controls, and had weaker phase-locking factors and gamma-synchronization predominantly in right parietal cortex.

**Conclusions:**

Our findings demonstrate that untreated patients with schizophrenia exhibit aberrant mirror neuron system function based on the right inferior parietal cortex, which is characterized by dysfunction of gamma-synchronization in the right parietal lobe during observation of biological motion.

## Introduction

Schizophrenia is characterized by deficits of social interaction. Patients exhibit impairment of social adaptation and functioning that results in social isolations. This impairment is due to underlying cognitive deficits, as revealed by different levels of social cognitive tasks. Deficits of motion perception, especially defective perception of biological motion (BM), are among the most important and established features. Recent neuroimaging and behavioral studies of schizophrenia have provided evidence of abnormal neural mechanisms related to observation of BM [1], [2]. Perception of BM is essential for the recognition of facial expressions and gestures and for understanding the goals, intentions, and desires of other people. As a result of defective perception of BM, patients with schizophrenia misunderstand nonverbal communication with others, resulting in impairment of social interaction [3]–[5].

The neural network subserving perception of BM is thought to consist of visual areas and the “mirror neuron system” (MNS) [6]. These neural systems respond during execution as well as recognition of actions, and were originally discovered by performance of single-cell recordings in the macaque monkey ventral premotor cortex (area F5) during the 1990s [7]–[9]. Subsequently, neurons with similar properties were found in various parts of the human cortex, including Broca's area, the premotor cortex, the superior temporal sulcus, and the rostral inferior parietal lobule (area PF) [10]–[12].

It has recently been shown that various cognitive dysfunctions associated with psychosis have included the disorders of emotion recognition, theory of mind, and affective responsiveness, and also have involved the dysfunction of the MNS [13]. In addition, the specific function of each cortical region implicated in MNS is suggested to be correlated with particular psychological manifestation. For example, “theory of mind”, that is the fundamental function in interacting each other in social context, could be associated with the neural activities in temporo-parietal junction [14]. Specifically, one of the first rank syndrome of schizophrenia, that is “misattribution of agency” in which the patients mistakenly attribute certain self-generated thoughts and actions to external sources [15], could be related with right parietal dysfunction. Therefore, the right parietal region involved in MNS is an attractive target for research, since putative dysfunction of this region could give rise to the pathological experiences commonly seen in patients with schizophrenia, bridging the wide range of signs, symptoms and the regional neural functions in these patients. Thus, we hypothesized that patients with schizophrenia could show the disturbance of MNS particularly in the right parietal activation in this magnetoencephalography (MEG) experiment, and predicted aberrant magnetic components specifically at high-frequency responses measured through fast Fourier transformation (FFT) and time-frequency power representations (TFR).

In this study, we examined whether antipsychotic-free patients with schizophrenia exhibit abnormalities of the MNS during observation of jaw movement. The high spatial and temporal resolution of MEG enabled detailed analysis, including assessment of gamma power and synchronization (both of which have been shown to be abnormal in patients with schizophrenia) as well as suppression as an index of MNS activity. This is the first comprehensive study of the MNS in patients with antipsychotic-free schizophrenia.

## Materials and Methods

### Subjects

Fifteen antipsychotic-free patients with schizophrenia ranging in age from 19 to 43 years (mean age = 33.4 years [SD = 6.6]; eight women) and 15 sex-, age-, and education-matched normal controls ranging in age from 20 to 42 years (mean age = 32.7 years [SD = 5.6]; seven women) (two-tailed unpaired *t*-test for age: p = 0.77; χ^2^ test for gender: p = 0.57) participated in this study. The never-medicated patient group consisted of six patients (four women) with their first episode and two patients (one woman) with recent-onset disease who had never been given antipsychotic drugs, while the seven medication-free patients (three women) had previously given one or two classes of antipsychotic drugs that had been stopped at least six months prior to this study. All patients met the DSM-IV-TR [16] diagnostic criteria for schizophrenia on the basis of interviews performed by two psychiatrists. The diagnosis was confirmed with the SCID-I interview (Structured Interview for DSM-IV) [17].

The characteristics of the patients, including the evaluations of clinical symptoms with the Positive And Negative Syndrome Scale (PANSS), are displayed in Table 1. All subjects from both groups had normal or normal-corrected visual acuity, and all were right-handed according to the Edinburgh Scale [18]. Prior to the study, all subjects were fully informed about MEG recording and all gave written informed consent. In addition, two senior clinical psychiatrists confirmed that all subjects with schizophrenia had abilities to consent in participating to the examination. In case of the patients under 20 years old, legal representatives gave written informed consent (subject 2 from her mother). This study was approved by the ethics committee of Tokyo Dental College in accordance with the Declaration of Helsinki protocols.

**Table 1:** Characteristics of the subjects.

Subject Number	Medication	Schizophrenic Subtype	Chronicity	Gender	Age on Exp. Day (years old)	Age of Onset (years old)	Duration of illness (months)	Duration of untreated psychosis (months)	Number of previous hospitalizations (times)	Marital	Professional	Educational status (years)	Past Medication	PANSS on Exp. Day				GAF on Exp. Day
														Positive	Negative	General	Total Score	
1	Antipsychotic-Naïve	Paranoid	First-episode	Female	31	31	1	1	0	Never married	Clerk of the trading company	14		16	9	36	61	25
2	Antipsychotic-Naïve	Paranoid	First-episode	Female	19	19	1	1	0	Never married	Student of the university (Department of law)	13		16	9	24	49	45
3	Antipsychotic-Naïve	Paranoid	First-episode	Male	35	34	2	2	0	Married	Public official	16		20	15	34	69	25
4	Antipsychotic-Naïve	Paranoid	First-episode	Female	23	23	2	2	0	Never married	Sales clerk of the tailor	14		11	11	22	44	55
5	Antipsychotic-Naïve	Paranoid	First-episode	Male	28	28	1	1	0	Never married	Office worker of the stationery manufacturers	16		13	11	25	49	45
6	Antipsychotic-Naïve	Paranoid	First-episode	Female	43	43	3	3	0	Married	Dentist	18		14	11	21	46	45
7	Antipsychotic-Naïve	Paranoid	Recent-onset	Female	32	32	9	9	0	Married	Housewife	14		24	21	56	101	15
8	Antipsychotic-Naïve	Disorganized	Recent-onset	Male	32	24	86	86	0	Never married	None (withdrawal from graduate school)	18	Etizolam 1 mg / day	19	33	43	95	20
9	Antipsychotic-Free	Paranoid	Chronic (recurrence)	Male	41	28	166	10	0	Previously married	Previously worked at real estate agent	16	Perphenazine 4 mg / day or Perospirone 4 mg / day	21	21	33	75	30
10	Antipsychotic-Free	Paranoid	Chronic (recurrence)	Female	31	29	16	9	0	Never married	Reception of the department store	16	Quetiapine 150 mg / day or Risperidone 1 mg / day	14	18	21	53	45
11	Antipsychotic-Free	Paranoid	Chronic (recurrence)	Male	34	22	143	18	5	Never married	Previously worked at sushi restaurant	12	Fluphenazine decanoate 50 mg / month	20	18	40	78	35
12	Antipsychotic-Free	Disorganized	Chronic (recurrence)	Male	39	21	209	19	1	Never married	None (drop out from the college)	15	Bromperidol 6 mg / day	22	34	52	108	15
13	Antipsychotic-Free	Paranoid	Chronic (recurrence)	Female	35	31	45	8	2	Never married	Pianist	16	Haloperidol 0.75 mg / day	20	17	32	69	35
14	Antipsychotic-Free	Paranoid	Chronic (recurrence)	Female	40	29	128	10	1	Never married	None (eight years hospitalization)	12	Olanzapine 20 mg / day	25	21	45	91	20
15	Antipsychotic-Free	Paranoid	Chronic (recurrence)	Male	38	20	211	20	0	Never married	None (used to be a student)	14	Zotepine 100 mg / day or Risperidone 1mg /day	18	14	43	75	25

Normal Controls								
Subject Number	Left				Right			
	MT / V5	STS	IPC	PMC	MT / V5	STS	IPC	PMC
1	164	232	250	285	164	232	250	285
2	171	230	238	335	171	230	238	335
3	145	228		290	145	228		290
4	146	221		275	146	221		275
5	161		255	288	161		255	288
6	164		270	299	164		270	299
7	152		249	278	152		249	278
8	127		238	290	127		238	290
9	157		242	279	157		242	279
10	145		249	290	145		249	290
11	164		267	301	164		267	301
12	178		254	280	178		254	
13	163		243		163		243	
14	141		247		141		247	
15	169		277		169		277	
Mean	156.5	227.8	252.2	290.8	156.5	227.8	252.2	291.8
S.D.	13.54	4.79	12.27	16.06	13.54	4.79	12.27	16.46

Schizophrenia Patients								
Subject Number	Left				Right			
	MT / V5	STS	IPC	PMC	MT / V5	STS	IPC	PMC
1 (SC-Naïve)	163		252	291	163		252	291
2 (SC-Naïve)	162	232			162	232		
3 (SC-Naïve)	135		250		135		250	
4 (SC-Naïve)	146		258		146			
5 (SC-Naïve)	137		288		137			
6 (SC-Naïve)	180				180			
7 (SC-Naïve)	162				162			
8 (SC-Naïve)	179				179			
9 (SC-Free)	141	227		285	141	227		
10 (SC-Free)	179		239		179		249	311
11 (SC-Free)	137		255		137			
12 (SC-Free)	151				151			
13 (SC-Free)	154				154			
14 (SC-Free)	127				127			
15 (SC-Free)	160				160			
Mean	154.2	229.5	257	288	154.2	229.5	250.3	301
S.D.	17.07	3.54	16.52	4.24	17.07	3.54	1.53	14.14

Fifteen normal controls and 15 antipsychotic-free schizophrenia patients participated in this study. Eight of the 15 patients were antipsychotic-naïve patients, while seven were antipsychotic-free patients with a history of using one or two antipsychotic drugs that had been terminated at least six months prior to this study. The profile includes the age on the day of study (mean = 33.4), age of onset of schizophrenia (mean = 27.6), duration of untreated psychosis (mean = 13.3 months), and educational status (mean = 14.9 years). The mean duration of illness for all patients was 68.2 [SD = 81.3] months (never-medicated patients = 13.1 [SD = 29.6], medication-free patients = 131.1 [SD = 75.8]). The mean total score of the Positive And Negative Syndrome Scale (PANSS) on the experimental day was 70.9 [SD = 20.8] with the mean positive score of 18.2 [SD = 4.1], negative score of 17.5 [SD = 7.7] and general score of 35.1 [SD = 11.2]. Also the mean score of global assessment of functioning (GAF) was 32.0 [SD = 12.6].

### Experimental paradigm

Prior to the study, each subject was seated inside a magnetically shielded room, while relaxing with their eyes open and fixed on a cross in front of them (Rest condition). This condition was employed as a control for frequency analysis. Spontaneous cortical activity was initially recorded for about 5 minutes in the Rest condition, after which subjects performed to the experimental paradigm.

**Figure 1:**
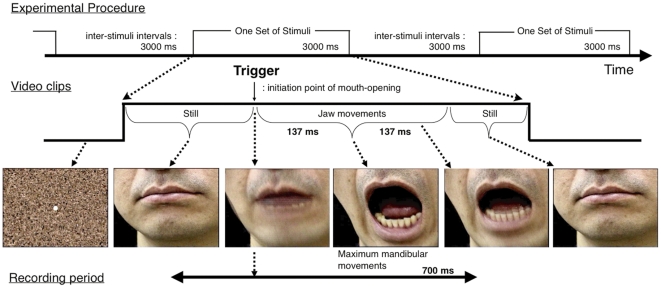
Sequence of video clips. In the BM condition, subjects were instructed to observe mouth-opening movements performed by another individual. Video clips contained 274-ms-long movements over a total of 3,000 ms in one set and were presented silently with an inter-stimulus interval of 3,000 ms. Triggers were provided at the initiation of mouth opening.

Each subject was instructed to carefully observe a set of pre-recorded video clips that showed line-symmetrical mouth opening movements of another individual (BM condition, i.e., observation of BM). These videos displayed mouth opening movements once, which lasted for 274 ms during a 3,000 ms to be one set, with the inter-stimulus interval was 3,000 ms. Video clips were projected onto a screen in front of the subject, subtending 3 degrees of visual angle. Figure 1 presents an example of video clips shown in the BM condition. In order to hold the subject's attention, deviant mouth movements (catch trials: holding a spoon in mouth/eating a peach/narrowing of the mouth) were occasionally presented (12% of all presentation), but the data obtained with such movements were excluded. Participants were instructed to look at the center of the screen and to press a button with their right thumb when they saw a spoon being held in the mouth (2% of all presentations). Data on 100 presentations were averaged off-line from about 120 to 130 presentations in total.

Event-related magnetic signals were collected and averaged using time-locked trigger pulses generated with a photo sensor in front of the projector. The sensor detected changes in the luminosity of a small square projected beside the visual stimulus, which appeared at the initiation of mouth opening. Subjects could not see this square, which was projected at trigger onset. After completion of the experimental paradigm, subjects were asked to imitate the mouth-opening movements that had been presented to ensure that they understood and could perform the same motions.

### MEG recording

Brain magnetic signals were measured with a whole-scalp 306-channel neuromagnetometer equipped with 204 planar gradiometers and 102 magnetometers (Vectorview, Elekta Neuromag Co., Helsinki, Finland) [19]. The exact position of the head with respect to the sensors was determined by measuring magnetic signals produced by indicator coils placed at known sites. The locations of each coil was determined by using a 3D digitizer (Isotrak, Vermont, USA) to enable alignment of the MEG and MRI coordinate systems. Head MRI was performed with a 1.5-T Siemens Symphony system (Erlangen, Germany). MEG responses were recorded using a 0.1–200 Hz band-pass filter and were digitized at a sampling rate of 997 Hz. About 100 artifact-free single responses were averaged off-line, with epochs in which signals exceeded 1500 fT/cm or were generated by coarse movements (e.g., head motion) being excluded from averaging.

### Data analysis

The continuous MEG responses were digitally low-pass filtered with 140 Hz cutoff using a half amplitude Gaussian filter, and averaged off-line with the time-locked trigger pulses. The analysis period was set at 700 ms, from 100 ms prior to 600 ms after the trigger pulse (initiation of mouth-opening in each video clip). The mean amplitude from -100 ms to 0 ms was served as the baseline for each channel. Each averaged response was analyzed, with the grand average waveform and root-mean-square (RMS; the square root of the sum of squared fT/cm values) being calculated from the 204 planar gradiometers. In addition, isocontour maps were constructed from the data at selected time points by the method of minimum norm estimates [20].

The sources of the magnetic fields were modeled as equivalent current dipoles (ECDs) based on the signals from the 306 channels for which the three-dimensional locations, orientations, and current strengths were estimated. A spherical head model was adopted, based on the MRI data obtained for each subject [21].

The ECDs that best explained the most dominant signals were first determined by least-squares analysis that identified a subset of channels over the areas where magnetic fields were visually detected. Goodness-of-fit (G/%) was also calculated. Only ECDs with a G/% of more than 85% were accepted for further analysis. Thereafter, analysis was extended to the entire period and to all channels, using a multi-dipole model. The measured signals explained by the model were extracted with Signal Space Projection [22], followed by a search for additional sources in the response of the residual waveforms. The validity of the multi-dipole model was evaluated by comparing the measured signals with responses predicted by the model. Finally, the ECDs obtained through this procedure were superimposed on the subject's MRI data by alignment of the MEG and MRI coordinate systems.

Raw MEG data obtained in the BM condition were subjected to fast Fourier transformation (FFT), over the frequency range of 1 to 49 Hz and the time window of 0 to 1,000 ms after the trigger onset in each subject. Data from the Rest condition were also averaged by FFT with time-step triggers every 1,024 ms. FFT power spectra within the range from 1 to 50 Hz were superimposed and their peak amplitudes and latencies were compared between the conditions (Rest or BM) and the subject groups (normal controls or patients). Peak amplitudes of RMSs in FFT windows were selected and calculated for two frequency bands, which were around 10 Hz (the peak of alpha band oscillations; ABO) and from 25 to 40 Hz (the peak of gamma band oscillations; GBO).

To quantify evoked responses in the high frequency range, i.e. phase-locking factors (PLF) and event-related gamma-synchronization (ERS), time-frequency power representations were obtained by a method based on Morlet wavelets (http://neuro.hut.fi/~tanzer/d4d/). For this transformation, the MEG signals were convoluted by complex Morlet wavelets with a Gaussian shape in the time and frequency domains to obtain those providing the best compromise for time and frequency resolution. The advantage of this method compared to short-time Fourier transformation is the adaptation of time resolution for each frequency band.

To detect evoked high-frequency activity, wavelet transformation of the average evoked responses was performed. From this time-frequency representation, a mean time-varying power value was computed between 8 and 60 Hz, with a time window of 800 ms that started 100 ms before the trigger (−100 to 700 ms), permitting analysis of the latency of the evoked high-frequency response.

Statistical examination of the differences in peak amplitudes was done for FFT windows across both conditions and both groups of subjects using a 2 × 2 × 2 analysis of variance (ANOVA) design with the condition (rest or BM) and the frequency band (ABO or GBO) as within-subject factors and the group as the between-subject factor (normal controls or schizophrenia patients). Pearson's correlation coefficients were also calculated between the PANSS scores and RMS components (numbers and amplitudes) with a significant level of p<0.05. Post-hoc comparison of mean values was done with Tukey's HSD test when ANOVA revealed a significant difference with p<0.001 being considered significant.

## Results

### Behavioral Data

No differences between the normal controls and schizophrenia patients were identified with respect to the detection rate (100% in both groups), the response time for catch trials (spoon in mouth) (two-tailed unpaired *t*-test for the response time: p = 0.92), and performance of mouth-opening movements.

### Waveform and RMS values – Biological Motion

From the waveforms of 204 channels and calculated RMS values, three components were observed in both hemispheres within the time window from -100 to 600 ms in all normal controls. The peak latencies of these components were about ∼160 ms (M1), 250 ms (M2), and 330 ms (M3) after the trigger.

**Figure 2:**
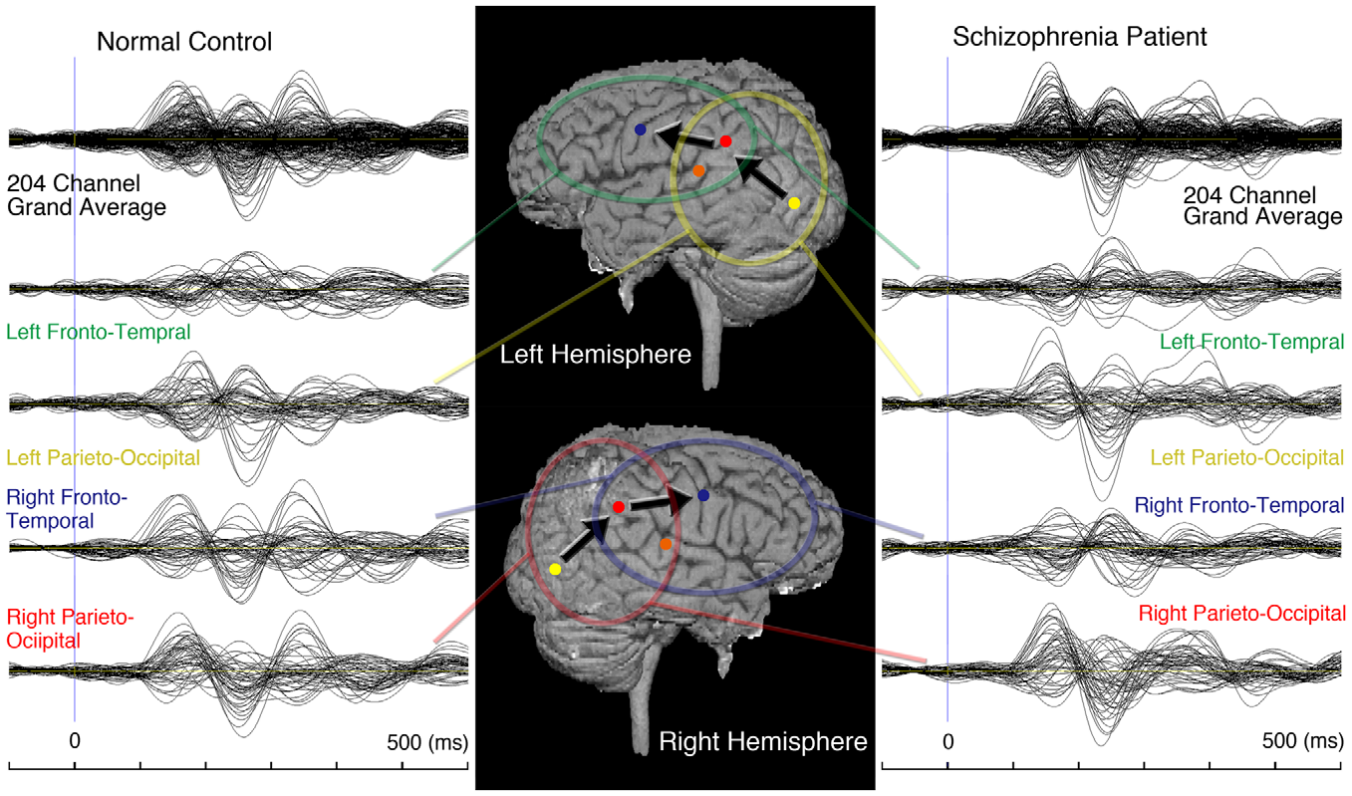
Sequential activation at the brain surface. Left and Right panels show grand average waveforms (filtered between 1 and 24 Hz with a time window from −100 to 600 ms) measured in a representative normal control (Left) and a representative schizophrenia patient (Right). In order from the top are the 204-channel grand average and the regional (50–52 gradiometers) averages for the left fronto-temporal (green), left parieto-occipital (yellow), right fronto-temporal (blue), and right parieto-occipital (red) areas corresponding to each region in the center panel, which depicts estimated ECDs at the brain surface. Most normal controls exhibited MT/V5 (yellow dot), IPC (red dot)/STS (orange dot), and PMC (blue dot) activation bilaterally, with a total of six areas of activation, while schizophrenia patients had fewer areas of cortical activation. In waveforms from the parieto-occipital regions, fewer components are identified in the schizophrenia patient (Right panel), in contrast to three bilateral components in the normal control (Left panel).

Figure 2 (left) shows grand average waveforms for normal control 1, while the right side shows such waveforms for schizophrenia patient 3. The top panels show the 204-channel grand average waveforms. From the upper to second below panels, regional grand average waveforms are shown for the channels located in the left fronto-temporal, left parieto-occipital, right fronto-temporal, and right parieto-occipital regions, respectively. These waveforms were superimposed and were obtained from 50 to 52 gradiometers located over each region. In normal control 1, three major components can be seen in both hemispheres, which are M1 (160 ms), M2 (250 ms), and M3 (340 ms).

Corresponding to the results of waveform analysis, three RMS components were identified in both hemispheres at the same latencies. Schizophrenia patients tended to display fewer components than normal controls, which were more prominent in right hemispheres. For the identified components, the latencies were almost the same as in the normal controls (M1 = 160 ms, M2 = 250 ms, and M3 = 330 ms). Differences between the controls and patients in the amplitudes of M2 and M3 were most marked in the right parieto-occipital region. In schizophrenia patient 3 (Figure 2; right panel) the bilateral M3 components were weaker compared with those in normal control 1 (Figure 2; left panel). Statistical analysis demonstrated a significant effect of the numbers (F(6,168) = 11.0, p<0.001) and amplitudes (F(2,56) = 18.5, p<0.001) of RMS components by groups. Post-hoc t-test showed that the amplitudes of M2 (t(28) = 3.89, p<0.0006) and M3 (t(28) = 3.92, p<0.0005) in the right parieto-occipital region were significantly weaker in patients than controls. The number of RMS components measured in 204 channels (t(28) = 3.76, p<0.001) in the right hemisphere (t(28) = 5.26, p<0.0001) were decreased in patients than controls, but not decreased in the left hemisphere (t(28) = 3.15, p<0.0038). Moreover, the negative score on PANSS were negatively correlated with the amplitudes of M2 (ρ = −0.539, p<0.038), M3 (ρ = −0.627, p<0.012). The total score of PANSS were significantly correlated negatively with the amplitudes of M3 (ρ = −0.534, p<0.040) within the schizophrenia patients.

### Source modeling - Biological Motion

Corresponding to the three magnetic components, a maximum of eight equivalent current dipoles (ECDs) were estimated in both hemispheres. Among the time-varying multi-dipole models [21], [22], six- to eight-dipole models provided the most reasonable solutions to the signals. In these models, ECDs were estimated in the bilateral MT/V5 regions at latencies of around 160 ms.

For the second components around 250 ms, ECDs were estimated to be located in the parietal cortex (IPC) and/or posterior superior temporal sulcus (STS). Finally, ECDs in the maxillofacial region of the primary motor cortex (PMC) were estimated at latencies around 300 ms in both the controls and the patients. Sequential activation of the cortex is shown schematically in Figure 2 (middle panel), which presents ECDs on a brain surface view for representative normal control 1.

Table 2 shows the latencies of ECDs in each subject. Missing data means an inappropriate location or insufficient amplitude of the ECDs. Only eight patients with schizophrenia exhibited IPC or STS activation, whereas all of the normal controls had the same regional activation patterns. Decreased activation of the PMC was noted in the schizophrenia patients, with activation only being seen in three patients (20%) versus 12 normal controls (80%). Fewer ECDs were appropriately estimated in the right hemisphere (total of 22 in patients and 43 in controls) as compared to the left (total of 25 in patients and 44 in controls) as was shown in Table 2. For each ECD, the source was stronger in the left hemisphere than the right hemisphere among both controls and patients. Statistical analysis revealed significant main effect of the source strengths of ECDs by those hemisphere (F(1,62) = 41.2, p<0.001). Post-hoc test demonstrated that the source strengths of ECD estimated in left hemispheres were significantly stronger than those estimated in right hemispheres (t(62) = 6.42, p<0.001).

**Table 2:**
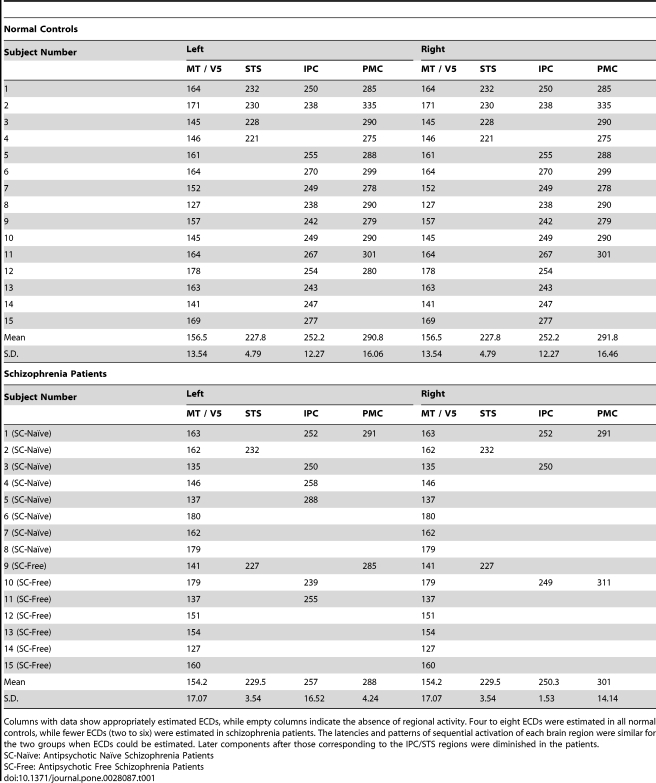
Latencies of estimated ECDs in each region.

Columns with data show appropriately estimated ECDs, while empty columns indicate the absence of regional activity. Four to eight ECDs were estimated in all normal controls, while fewer ECDs (two to six) were estimated in schizophrenia patients. The latencies and patterns of sequential activation of each brain region were similar for the two groups when ECDs could be estimated. Later components after those corresponding to the IPC/STS regions were diminished in the patients.

SC-Naïve: Antipsychotic Naïve Schizophrenia Patients

SC-Free: Antipsychotic Free Schizophrenia Patients

These waveform, RMS, and ECD data indicated that patients with schizophrenia had deficiencies of sequential cortical activation, which seemed to originate from insufficient activation of the M2 component arising from IPC or STS cortical activity.

### Fast Fourier Transformation

**Figure 3:**
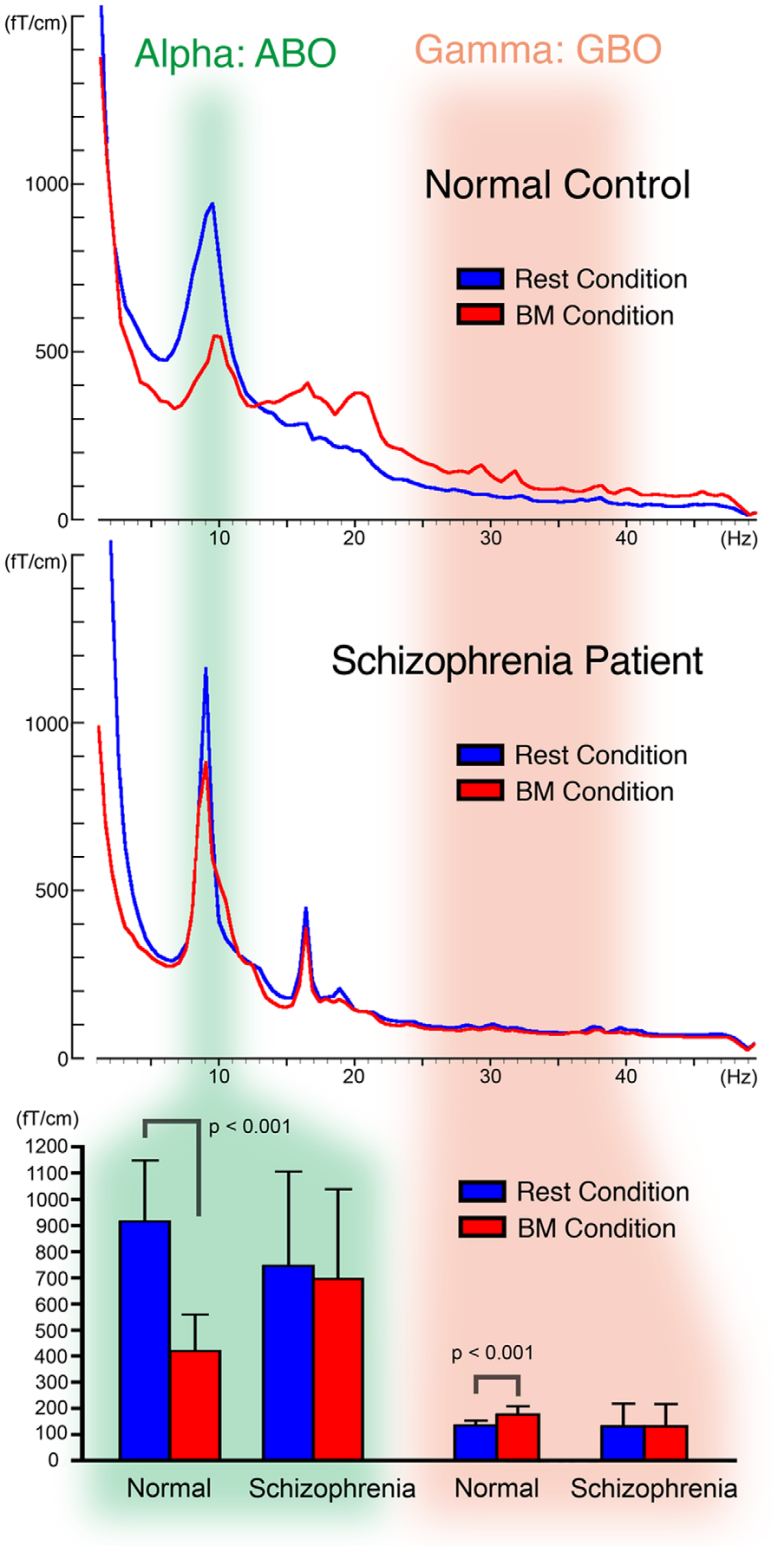
FFT window and peak amplitudes of alpha band oscillation (ABO) and gamma band oscillation (GBO). The upper and middle panels show averaged FFT distributions between 1–50 Hz for normal controls and schizophrenia patients, respectively. The lower panel shows the mean amplitudes and standard deviations (error bars) of ABO (around 10 Hz; green highlight) and GBO (25 to 40 Hz; orange highlight) in each group. Blue lines and bars denote the Rest condition, while Red lines and bars denote observation the BM condition. In normal controls, the peak amplitude of ABO was suppressed significantly (p<0.001) and GBO was amplified significantly (p<0.001) in the BM condition compared with the control condition. On the other hand, there were no differences between the two conditions for schizophrenia patients. Increased and scattered GBO activity in the Rest condition was also noted in the schizophrenia patients.

By calculating fast Fourier transformation (FFT), all subjects exhibited one peak (around 10 Hz) in the Rest condition, while two peaks (10 and 25∼40 Hz) were seen in the BM condition. A typical change of FFT between both conditions in normal controls was suppression of the peak around 10 Hz (ABO) and an increase of the peak at 25∼40-Hz (GBO). Figure 3 shows FFT windows from normal control 1 (upper panel) and schizophrenia patient 3 (middle panel), as well as the peak amplitudes of the two groups (lower panel) under both the Rest condition (blue lines and bars) and the BM condition (red lines and bars). In normal controls, ABO peaks (green highlight) were suppressed while GBO peaks (orange highlight) were amplified under the BM condition relative to the Rest condition. In contrast, patients with schizophrenia exhibited nearly the same amplitudes under both conditions. In normal controls, the mean (± S.D.) ABO amplitude obtained as the sum of the FFTs for 204 channels was 915.1 ± 232.5 fT/cm under the Rest condition, with suppression to 419.7 ± 139.4 fT/cm under the BM condition. In schizophrenia patients, the mean ABO amplitude under the Rest condition (745.1 ± 360.3) was similar to that under the BM condition (695.6 ± 342.2). On the other hand, normal controls showed an increase of mean GBO from 133.6 ± 18.7 to 175.5 ± 30.3 during observation of BM, while the two values were almost the same in schizophrenia patients (130.4 ± 85.7 for Rest to 129.2 ± 86.0 for BM).

Statistical analysis demonstrated a significant main effect of the condition (F(1, 28) = 99.1, p<0.001) and a significant condition-by-group interaction (F(1, 28) = 63.2, p<0.001). Post-hoc two-tailed, two-sample *t*-tests demonstrated significant suppression of ABO in normal controls (t(14) = 11.00, p<0.001), but not in schizophrenia patients (t(14) = 3.07, p = 0.0083). GBO was significantly amplified in normal controls (t(14) = 5.24, p<0.001), but not in schizophrenia patients (t(14) = 0.16, p = 0.8775).

### Time Frequency Representations (TFR)

**Figure 4:**
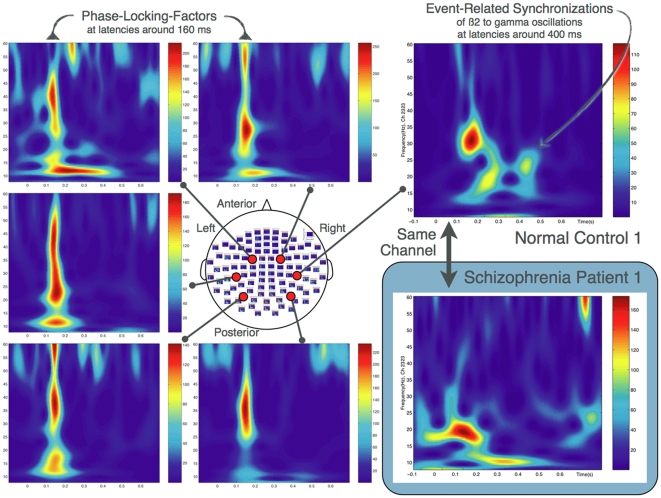
Time frequency representations (TFR) for representative control 1 (and insets for patient 1). For almost all channels, phase-locking factors (PLF) were observed at latencies of 160 ms throughout the frequency range. Event-related gamma-synchronization (ERS) was also observed in the normal control, mainly in the temporal to parietal regions. The schizophrenia patient exhibited almost the same frequency and latency distributions, except for weaker PLF and ERS predominantly in the right parietal region. Inset, shows TFR of patient 1 from the same channel located in right parietal region, exhibiting absence of these synchronizations.

Moreover, we calculated time-frequency representations (TFR) and plotted the data on the basis of Morlet wavelets for channels that were considered representative of each region. Figure 4 depicts TFRs calculated from the channels located in the bilateral occipital, parietal, and temporal regions of normal control 1 (the inset displays TFR data from the same channel in the right parietal region of schizophrenia patient 3). In all normal controls, phase-locking factors (PLF) were identified at latencies of around 160 ms throughout the frequency range in almost all channels. In addition, event-related gamma-synchronization (ERS) was commonly observed at latencies of around 400 ms, being most evident in the bilateral temporo-parietal regions (right > left). Schizophrenia patients had almost the same frequency and latency patterns as the normal controls in all channels, except for those in the right parietal region. In this region, the schizophrenia patients tended to have weaker PLF and ERS (see File S1).

## Discussion

The main findings of this magnetoencephalography (MEG) study were as follows. While observing biological motion (BM), patients with schizophrenia exhibited fewer components of both waveforms and equivalent current dipoles (ECD) than normal controls, suggesting that they had aberrant brain activity resulting from dysfunction of the right inferior parietal cortex. They also lacked the changes of alpha band oscillations (ABO) and gamma band oscillations (GBO) seen in normal controls, and exhibited weaker phase-locking factors (PLF) and event-related gamma-synchronization (ERS), predominantly in the right parietal cortex. We thus demonstrated that subjects with schizophrenia had a deficit of mirror neuron system (MNS) function in the right inferior parietal cortex, which was characterized by impaired of gamma oscillation in the right parietal lobe during observation of BM.

Previous studies have suggested that passive viewing of BM (particularly of goal-directed hand action and of mouth movement [23]) alone could activate various brain regions [24]–[26] that are thought to have a role in the human MNS. In agreement with these earlier studies, four to eight ECDs were estimated in both hemispheres. Cortical activation chains were located at MT/V5 (at a latency of 160 ms), the superior temporal sulcus (STS) (230 ms), the inferior parietal cortex (IPC) (250 ms), and the primary motor cortex (PMC) (300 ms), areas which were found to participate in the human MNS by earlier studies (Figure 2, Table 2).

In contrast, schizophrenia patients exhibited fewer components of their waveform, RMS, and ECD data, typically lacking right M2 components and having fewer ECDs in the IPC region, followed by absence of components for later brain activity (e.g. M3 and PMC) (Table 2). However, the earlier components (e.g. MT/V5) showed no differences of activation pattern between the two groups (Figure 2). Moreover, the overall and negative symptom severities were negatively correlated with the neural activations (M2 and M3). Thus, patients with schizophrenia exhibited loss of an entire chain of activation, presumably related to right parietal dysfunction, when the activation sequence was taken into account.

FFT analysis revealed that the peak amplitude of ABO was suppressed and that of GBO was amplified significantly in normal controls under the BM condition, whereas no differences of either frequency band (ABO or GBO) were found in schizophrenia patients (Figure 3).

It has been investigated that the “mu” wave (8–13 Hz), which were corresponding to the ABO in our study, were suppressed by observing actions in normal controls [27], but not suppressed in autism spectrum disorders [28] and schizophrenia [29]. Since “mu” suppression can be used as a selective measure of MNS function [30], the lack of ABO suppression in our patients with schizophrenia strongly suggests the existence of MNS dysfunction.

A number of other studies have also demonstrated an enhanced GBO response to a variety of cognitive tasks in healthy subjects [31], [32], with a contrasting decreased response in schizophrenia patients [33]–[35]. However, it is the synchronization of GBO that is of more interest for research [36], [37], since it appears to be impaired in schizophrenia. In fact, abnormal synchrony has been demonstrated in patients with schizophrenia performing a variety of cognitive tasks, such as perceptual binding and motor responses [38], though there has been no report concerning synchrony and MNS. Our present findings of lack of ABO suppression and decreased GBO amplification in schizophrenia patients strongly suggest dysfunction of synchrony and the MNS in schizophrenia.

These results of FFT analysis were based on summation of one-second intervals of the MEG responses. To specify each component affecting FFT data in the frequency and temporal domains, we then performed TFR analysis. Phase-locking factors (PLF) and event-related gamma-synchronization (ERS) were both identified in normal controls, whereas the schizophrenia patients had weaker activity, predominantly in the right parietal region. Moreover, TFR analysis revealed differences in the distribution of ERS between the two groups. Schizophrenia patients had weaker PLF amplitudes across all frequency bands and aberrant ERS, predominantly in the right parietal region (Figure 4). These findings suggest that patients with schizophrenia have an abnormality of the right parietal region that leads to MNS dysfunction.

The right parietal region has been considered as a candidate pathophysiological substrate for the signs and symptoms of schizophrenia. For example, disturbances of self-monitoring could result in typical subjective experiences of “misattribution of agency” [15]. Since the right inferior parietal lobe plays a crucial role in distinguishing one's own actions from those of others, dysfunction of this region could give rise to pathological experiences of this sort. Similarly, attribution of action to another agent has been shown to be associated with increased activity in the right inferior parietal lobe [39]–[42]. In patients with first rank schizophrenia syndrome, another study demonstrated that the experience of agency during production of an action involved the right inferior parietal lobule [43]. The authors suggested that there was a close connection between this cortical region and disturbance of agency, since the first rank syndrome should be a consequence of loss of the boundaries between self and others. The findings of the present study are consistent with these results, in suggesting involvement of the right inferior parietal lobule in disturbance of the sense of agency in patients with schizophrenia.

One limitation of this study was the small sample size (N = 30; 15 schizophrenia patients and 15 normal controls). There were also heterogeneous clinical features of the patients (schizophrenia subtype, chronicity, and past medications), which may make it difficult to interpret the relationship between neuroimaging data and symptoms. Previous studies have shown that the chronic course and active symptomatology of schizophrenia may lead to different responses depending on the patient. For instance, Lee et al. [44] demonstrated distinct patterns of abnormal gamma activity in relation to the symptom profile, i.e., reality distortion was associated with increased right synchrony, psychomotor poverty was related to reduced left synchrony, and disorganization was associated with widespread enhancement and delayed frontal synchrony. Future studies of these relationships using the present paradigm in a larger sample are warranted. An interesting point is whether a relationship exists between “misattribution of agency” and right parietal lobe function.

Despite these limitations, however, abnormal BM perception [1], [2] and gamma-synchronization [34], [45] found in the current study, which have been reported to be independently implicated in schizophrenia, and both factors converged on the right inferior parietal lobule, strongly support our hypothesis. We therefore propose that the present findings can be considered as traits of schizophrenia.

One influential hypothesis is that the psychopathology of schizophrenia is best understood in terms of abnormal interactions or integration between different cortical areas [46], [47], suggesting that this condition cannot be reduced to a mere parietal lesion syndrome. However, accumulating evidence suggests that the human brain contains specific computational systems that distinguish between self and others [48], [49]. Impairment of such a source monitoring system could lead to “contamination” of an individual's thoughts or feelings by those of others [50], [51]. Our findings strongly support a crucial role for the right parietal lobe in a source monitoring system and the existence of a correlation between disruption of this system and various pathological experiences in schizophrenia.

In antipsychotic-free schizophrenia patients, we found wide-ranging impairment of cortical activation related to BM perception, especially in the right parietal region. They also displayed alteration of gamma-synchronization in the frequency and temporal domains, which was found to originate from the right inferior parietal lobule. A dynamic view of the relation between these pathologies, which were previously thought to be independent in schizophrenia patients, is thus warranted.

## Supporting Information

File S1 **TFR for all channels from four representative subjects.** Examples of Time Frequency Representation (TFR) for all channels were shown in File S1. Full channels' TFR from representative four subjects (schizophrenia patient 1 and 2; normal control 1 and 2) were available with frequency window between 8 to 60 Hz of all gradiometer.
(PDF)
