# Supplementary material for: Magnetoencephalography Study of Right Parietal Lobe Dysfunction of the Evoked Mirror Neuron System in Antipsychotic-Free Schizophrenia
Source: PLoS One. 2011 Nov 22;6(11):e28087. doi: 10.1371/journal.pone.0028087 (PMC3222679; doi:10.1371/journal.pone.0028087)

File S1

<u>Supporting Information – Time Frequency Representations</u>

Figures below were the time-frequency representations from schizophrenia patient 1 and 2 and normal control 1 and 2 with frequency windows between 8 to 60 Hz of all gradiometer channels (anterior on upper, right on right).

In normal controls, phase-locking-factors (PLF) were observed strongly in bilateral occipital to parietal regions at the latencies around 160ms (i.e. MEG 2032/2033, MEG 2023/2022, MEG 2242/2243, MEG 1832/1833, MEG 2013/2012, MEG 2042/2043). Furthermore, event-related gamma-synchronizations (ERS) were observed in right parietal regions, which were more prominent in right hemispheres, at latencies around 400ms (i.e. MEG 2323/2322, MEG 2433/2432).

In contrast, schizophrenia patient elicited weaker responses than normal controls, which were more marked in right parietal regions (i.e. MEG 2323/2322, MEG 2433/2432), characterized by the lacks of PLFs and ERSs observed in normal controls.

TFR for all channels from normal control 1

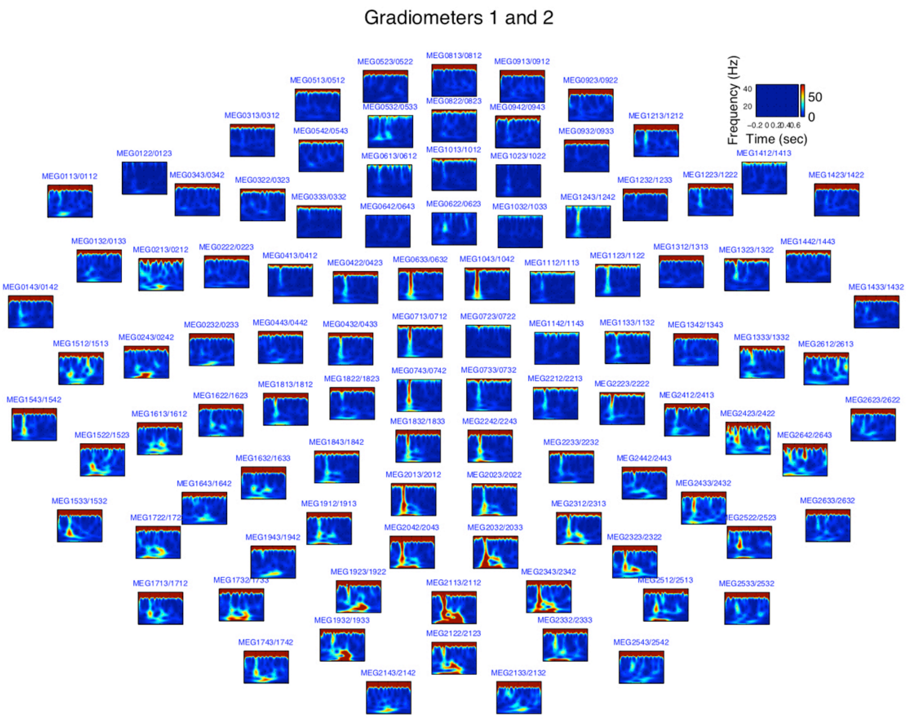

TFR for all channels from normal control 2

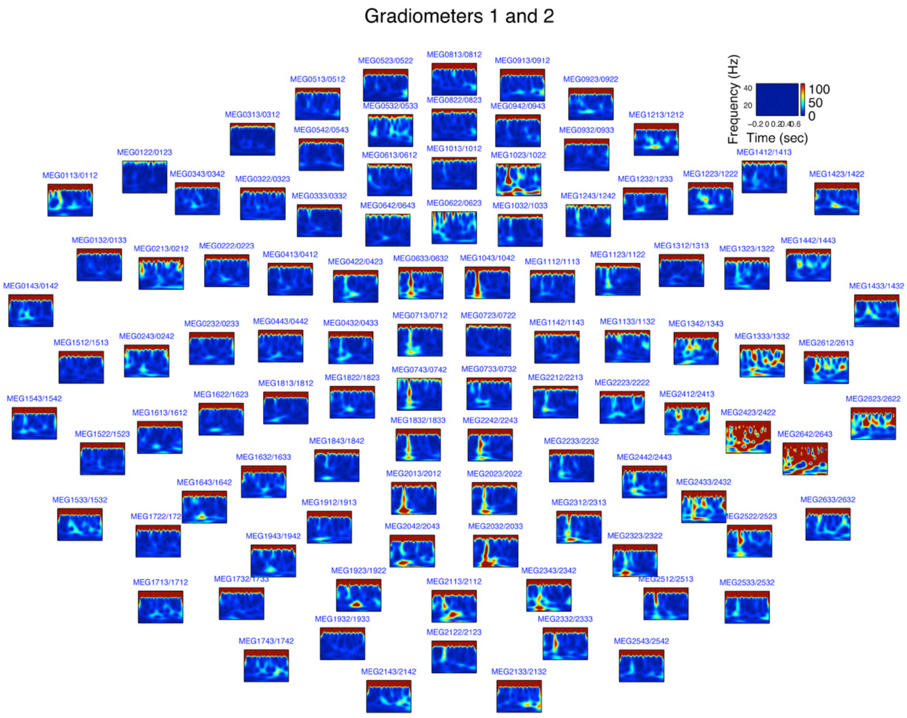

TFR for all channels from schizophrenia patient 1

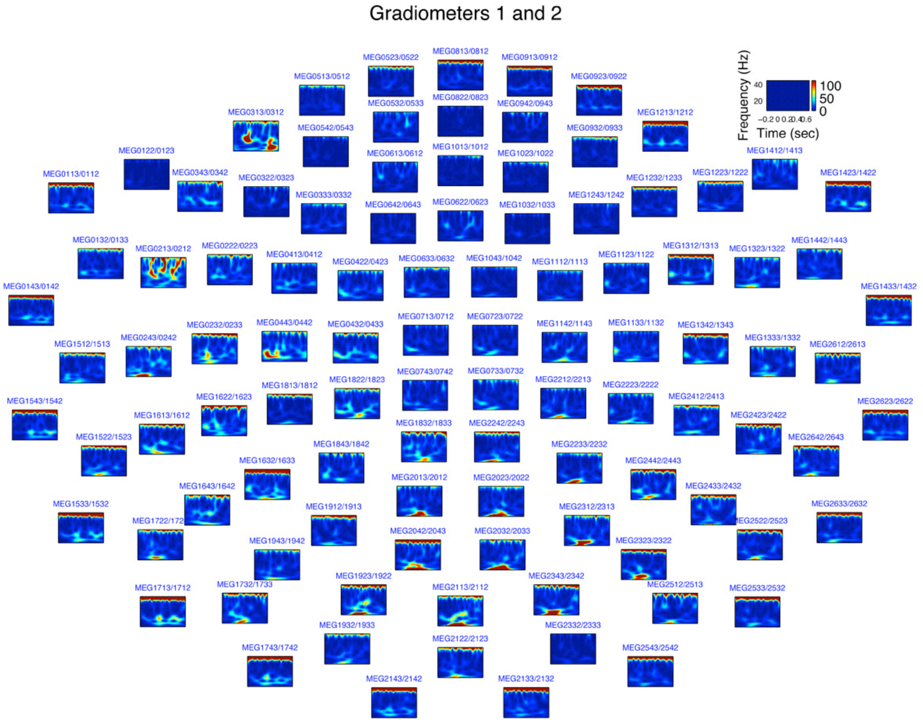

TFR for all channels from schizophrenia patient 2

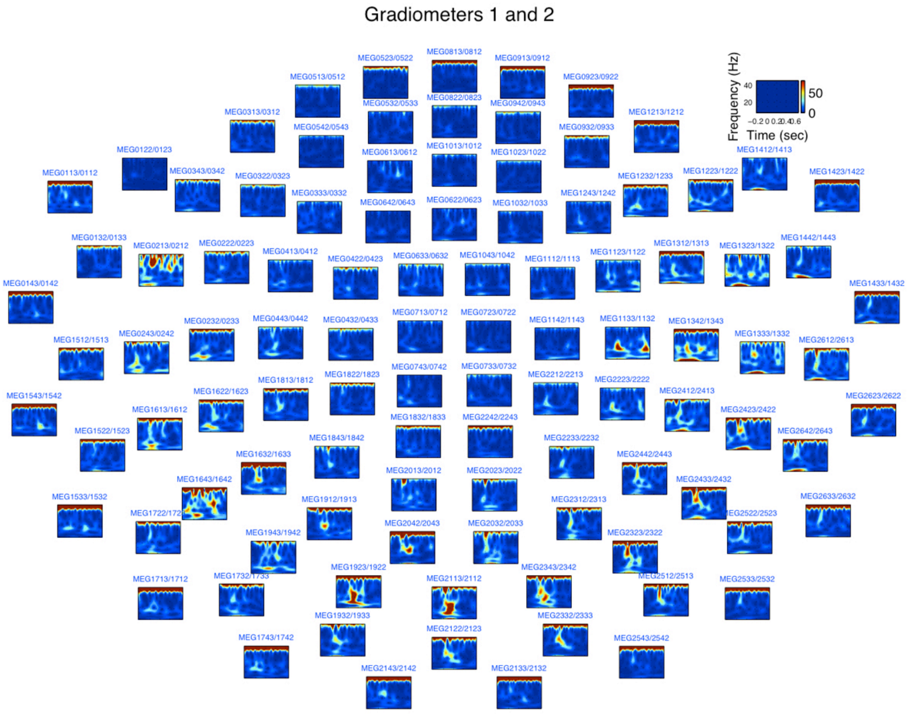

Supplement: File S1 — TFR for all channels from four representative subjects. Examples of Time Frequency Representation (TFR) for all channels were shown in File S1. Full channels' TFR from representative four subjects (schizophrenia patient 1 and 2; normal control 1 and 2) were available with frequency window between 8 to 60 Hz of all gradiometer. (PDF) [file pone.0028087.s001.pdf]
